# Limited phylogenetic overlap between fluoroquinolone-resistant *Escherichia coli* isolated on dairy farms and those causing bacteriuria in humans living in the same geographical region

**DOI:** 10.1093/jac/dkab310

**Published:** 2021-08-27

**Authors:** Oliver Mounsey, Hannah Schubert, Jacqueline Findlay, Katy Morley, Emma F Puddy, Virginia C Gould, Paul North, Karen E Bowker, O Martin Williams, Philip B Williams, David C Barrett, Tristan A Cogan, Katy M Turner, Alasdair P MacGowan, Kristen K Reyher, Matthew B Avison

**Affiliations:** 1 School of Cellular & Molecular Medicine, University of Bristol, Bristol, UK; 2 Bristol Veterinary School, University of Bristol, Bristol, UK; 3 Department of Microbiology, Infection Sciences, Southmead Hospital, North Bristol NHS Trust, Bristol, UK; 4 Bristol Royal Infirmary, University Hospitals Bristol and Weston NHS Foundation Trust, Bristol, UK

## Abstract

**Background:**

Our primary aim was to test whether cattle-associated fluoroquinolone-resistant (FQ-R) *Escherichia coli* found on dairy farms are closely phylogenetically related to those causing bacteriuria in humans living in the same 50 × 50 km geographical region suggestive of farm–human sharing. Another aim was to identify risk factors for the presence of FQ-R *E. coli* on dairy farms.

**Methods:**

FQ-R *E. coli* were isolated during 2017–18 from 42 dairy farms and from community urine samples. Forty-two cattle and 489 human urinary isolates were subjected to WGS, allowing phylogenetic comparisons. Risk factors were identified using a Bayesian regularization approach.

**Results:**

Of 489 FQ-R human isolates, 255 were also third-generation-cephalosporin-resistant, with strong genetic linkage between *aac(6’)Ib-cr* and *bla*_CTX-M-15_. We identified possible farm–human sharing for pairs of ST744 and ST162 isolates, but minimal core genome SNP distances were larger between farm–human pairs of ST744 and ST162 isolates (71 and 63 SNPs, respectively) than between pairs of isolates from different farms (7 and 3 SNPs, respectively). Total farm fluoroquinolone use showed a positive association with the odds of isolating FQ-R *E. coli*, while total dry cow therapy use showed a negative association.

**Conclusions:**

This work suggests that FQ-R *E. coli* found on dairy farms have a limited impact on community bacteriuria within the local human population. Reducing fluoroquinolone use may reduce the on-farm prevalence of FQ-R *E. coli* and this reduction may be greater when dry cow therapy is targeted to the ecology of resistant *E. coli* on the farm.

## Introduction

Fluoroquinolones are an important class of antibacterial drugs, included in the WHO’s list of ‘Highest Priority Critically Important Antimicrobials’.^1^ They are extensively used to treat infections in humans, companion animals and farmed animals. These important medicines work by disrupting the activity of type II topoisomerases, causing the release of DNA that has double-strand breaks, leading to cell death.^2^

In addition to safety concerns about fluoroquinolone use in humans,^3^ bacteria such as *Escherichia coli* are increasingly becoming fluoroquinolone-resistant (FQ-R).^4^ Here, FQ-R is mainly caused by mutations altering the QRDRs of the primary target, DNA gyrase subunit A (GyrA), and the secondary target, DNA topoisomerase IV subunits (ParC) and, to a lesser extent, ParE.^5^^,^^6^ However, additional mechanisms can also contribute to FQ-R, including regulatory mutations leading to AcrAB-TolC efflux pump overproduction and expression of various plasmid-mediated quinolone resistance (PMQR) genes. For example, *qnr* genes, *oqxAB* efflux pump genes and *aac(6’)Ib-cr*, which encodes a mutated aminoglycoside-modifying enzyme.^5^^,^^7^

Because most FQ-R in *E. coli* results from multiple mutations in target enzymes and because PMQR genes cannot confer FQ-R alone, the spread of FQ-R *E. coli* typically involves vertical dissemination of resistant clones. Most commonly in humans, the widespread proliferation of an FQ-R subset of the ST131 complex,^8^ which is also frequently associated with third-generation-cephalosporin resistance (3GC-R).^9^ Additionally, the pandemic ST1193 group,^10^^,^^11^ which is only occasionally 3GC-R.^10^^,^^12^

In secondary care, the fluoroquinolone ciprofloxacin is commonly used as an oral switch following IV therapy for serious infections caused by Gram-negative bacteria.^13^ Accordingly, FQ-R is an important threat to human health and particularly when found in urinary *E. coli*, since a substantial proportion of sepsis has a urinary origin and *E. coli* is the leading cause of urosepsis.^14^ The use of fluoroquinolones in primary care in the UK has reduced in recent years, primarily due to changes in policies concerning prescribing for community urinary tract infections. For example, in the 1.5 million population centred on the city of Bristol, we recently demonstrated a 24% fall in dispensing of ciprofloxacin, by far the most used fluoroquinolone in primary care, between 2013 and 2016. We noted a commensurate reduction in FQ-R in community-origin urinary *E. coli* in the same region.^15^

Fluoroquinolones have also been extensively used for the treatment of companion^16^ and farmed^17^ animals. Usage has decreased in farmed animals in the UK over recent years^18^ and voluntary use restrictions were introduced in June 2018 that mean fluoroquinolones are only used as a last resort, backed up by susceptibility testing.^19^ There is still the possibility, however, that FQ-R *E. coli* that have been selected on farms might colonize humans and ultimately cause disease. There is also strong evidence that antibacterial-resistant *E. coli* are shared between companion animals and humans.^20^ Hence, considering FQ-R in humans through the lens of the One Health research framework may help obtain a wider picture of selection and transmission. Our primary aim was to test the hypothesis that FQ-R *E. coli* obtained in 2017–18 from dairy farms located within a 50 × 50 km region of South-West England were closely related to FQ-R human urinary *E. coli* collected in parallel from the same region, suggestive of sharing between these compartments. Whilst our similarly motivated studies of 3GC-R *E. coli* showed no evidence of recent sharing of isolates between dairy farms and humans,^21^^,^^22^ we considered that the clonal nature of FQ-R *E. coli* might give a different outcome.

## Methods

### Isolates

Collection of FQ-R *E. coli* from dairy farms in South-West England has been described previously.^23^ Briefly, 4145 samples were collected from faecally contaminated sites around 53 dairy farms (49 farms yielded FQ-R *E. coli*). Each farm was visited monthly between January 2017 and December 2018 to collect samples. FQ-R isolates were selected by plating processed samples onto Tryptone Bile X-Glucuronide (TBX) agar (Sigma–Aldrich, Dorset, UK) containing 0.5 mg/L ciprofloxacin, the EUCAST breakpoint.^24^ One FQ-R *E. coli* colony was picked from each plate if growth occurred. FQ-R urinary *E. coli* isolates were obtained from routine urine microbiology at Severn Infection Partnership, Southmead Hospital. Urine samples were submitted between September 2017 and December 2018 from 146 GPs located throughout Bristol and including coverage in Gloucestershire, Somerset and Wiltshire, a population of 1.5 million people.^15^ Bacterial identification was carried out using CHROMagar Orientation Medium (BD, GmbH, Heidelberg, Germany). Antibiotic susceptibilities were defined by disc testing using ciprofloxacin and ceftriaxone as indicators of FQ-R and 3GC-R, respectively, as interpreted according to EUCAST guidelines.^24^ FQ-R/3GC-R urinary isolates and FQ-R but third-generation-cephalosporin-susceptible (FQ-R/3GC-S) isolates were all subcultured onto TBX agar containing 0.5 mg/L ciprofloxacin to confirm FQ-R status.

### Ethics

Farmers gave fully informed consent to participate in this study. Ethical approval was obtained from the University of Bristol’s Faculty of Health Sciences Research Ethics Committee (ref. 41562). Ethical approval was not needed for the retention of human urinary isolates since these were anonymous and isolated for routine diagnostics.

### PCR, WGS and phylogenetic analysis

PMQR genes [*qnrA, qnrB, qnrC, qnrD, qnrS, aac(6’)-Ib-cr, oqxAB* and *qepA*] were identified by multiplex PCR as previously described.^25^ WGS was performed by MicrobesNG (https://microbesng.uk/) as previously described.^9^^,^^22^ Resistance genes and STs were assigned using ResFinder^26^ and MLST 2.0^27^ on the Centre for Genomic Epidemiology (http://www.genomicepidemiology.org/) platform. All WGS data have been deposited in the European Nucleotide Archive under project accession number PRJEB45949.

Phylogenetic analysis was carried out using the Bioconda environment^28^ on the Cloud Infrastructure for Microbial Bioinformatics.^29^ The reference sequences were ST131 isolate EC958 (accession* *=* *HG941718), ST744 isolate EC590 (accession* *=* *NZ_CP016182) and ST162 isolate W2-5 (accession* *=* *NZ_CP032989). Sequence alignments were with Snippy (https://github.com/tseemann/snippy). Maximum likelihood phylogenetic trees were constructed using RAxML, utilizing the GTRCAT model of rate heterogeneity and the software’s autoMR and rapid bootstrap.^30^^,^^31^ Trees were illustrated using Microreact.^32^

### Risk factor analysis

The risk factors fell into four categories: farm management, antibacterial usage (ABU), sample characteristics and meteorological. These categories, and the methods of data collection for each, are described in our recent wider risk factor study and its supplementary information.^23^ All code can be found at https://github.com/HannahSchubert1/OH-STAR-modelling-code. A random intercept Bayesian model with farm as a random effect was fitted using R with the BRMS (https://cran.r-project.org/web/packages/brms/index.html) package. The presence of FQ-R *E. coli* within a sample was the dependent variable. The remaining variables were all fixed effects and were split into two groups: ‘main effects’, which were the ABU variables (measured as mg per population-corrected unit) hypothesized to affect FQ-R *E. coli* presence, namely total fluoroquinolone usage, novobiocin usage and total third- and fourth-generation cephalosporin usage; and ‘regularized effects’, which were all the remaining variables.

For the main effects, uninformative priors (normal distribution with a mean 0, standard deviation 5) were used; for the regularized effects, a regularizing prior (horseshoe prior with a single degree of freedom)^33^ was used. The mean intercept was also given an uninformative prior (normal distribution with mean 0, standard deviation 5), while the standard deviation of the random effects was given the default prior for BRMS (a half-Student-t distribution with 3 degrees of freedom and a scale factor of 10). Four chains were sampled with 2000 iterations. The target acceptance criterion was increased from the default 0.8 to 0.98 to decrease the chance of sampling divergencies.

## Results

### Molecular epidemiology of FQ-R human urinary E. coli

A total of 489 FQ-R human urinary *E. coli* isolates were collected; 255 were also 3GC-R, while 234 were 3GC-S. According to PMQR gene multiplex PCR, 19 isolates harboured *qnr* genes (14 *qnrS* and 5 *qnrB*) and two carried *oqxAB*. Notably, 135 (52.9%) of the 3GC-R isolates but only 26 (11.1%) of 3GC-S isolates carried *aac(6’)Ib-cr* (χ^2^* *=* *96.7, *P *<* *0.0001).

**Figure 1.**
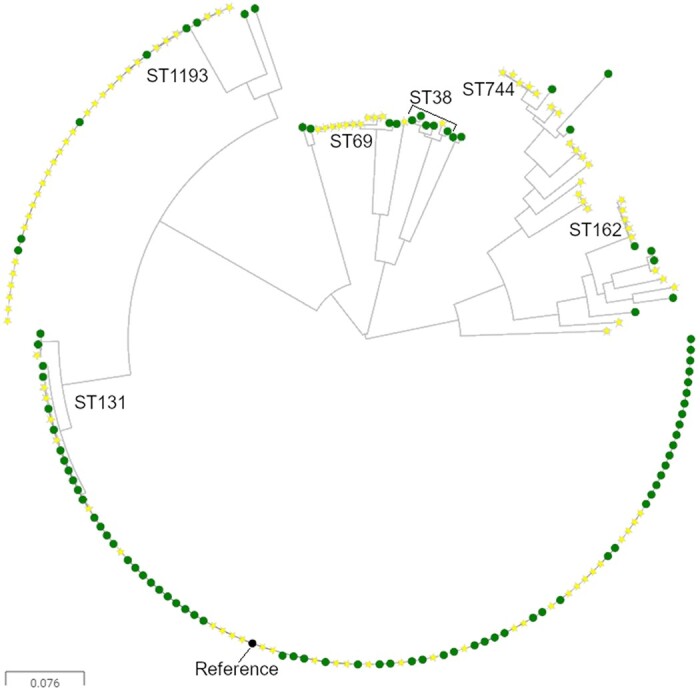
Maximum likelihood phylogenetic tree showing FQ-R urinary isolates. Green circles represent isolates that were also 3GC-R, whilst yellow stars represent isolates that were 3GC-S. Sequences were aligned against an ST131 reference (black node). STs have been indicated where more than five isolates from that ST were present. This figure appears in colour in the online version of *JAC* and in black and white in the print version of *JAC*.

WGS was performed for 188 FQ-R urinary isolates; 90 were 3GC-R and 98 were 3CG-S. Figure 1 shows a phylogenetic tree drawn based on core genome alignment for these isolates. As well as illustrating the dominance of ST131 (mainly 3GC-R, green markers) and ST1193 (mainly 3GC-S, yellow markers) this tree also shows that clusters of isolates fell into ST744, ST162, ST69 and ST38 (Figure 1). A total of 22 different combinations of QRDR mutations were identified in the 188 sequenced isolates. However, a majority (155/188) fell within three QRDR types, identified here as types A, B and C (Table 1). Isolates within common FQ-R clonal groups ST131 and ST1193 were dominated by QRDR types A and B, respectively, whilst QRDR type C was found in isolates from 13 diverse STs.

**Table 1.** Common QRDR mutation patterns identified within FQ-R human urinary *E. coli*

QRDR type	Associated ST	Amino acid sequence effect of mutations relative to wild-type strain (K12)	Number of isolates (188 total)
A	ST131	*gyrA* Ser83Leu, *gyrA* Asp87Asn, *parC* Ser80Ile, *parC* Glu84Val, *parE* Ile529Leu	90
B	ST1193	*gyrA* Ser83Leu, *gyrA* Asp87Asn, *parC* Ser80Ile, *parE* Leu416Phe	37
C	13 STs	*gyrA* Ser83Leu, *gyrA* Asp87Asn, *parC* Ser80Ile	28

WGS also confirmed the strong bias for *aac(6’)Ib-cr* gene carriage, with 39 (43.3%) 3GC-R isolates being *aac(6’)Ib-cr*-positive [versus only 4 (4.1%) of the 3GC-S isolates] (χ^2^* *=* *16.5, *P *<* *0.0001). We hypothesized that physical linkage of *aac(6’)Ib-cr* with an over-represented 3GC-R gene was driving this bias and Table 2 reports that carriage of *aac(6’)Ib-cr* was strongly associated with carriage of *bla*_CTX-M-15_. We thus further hypothesized that this was due to physical linkage of *bla*_CTX-M-15_ with the class 1 integron In*37*, carrying *aac(6’)Ib-cr*. Even given the difficulties of interpreting short-read WGS data, there was evidence in favour of this hypothesis. Of 11 isolates where the genomic positions of these loci could be definitively identified, in 3, In*37* and *bla*_CTX-M-15_ were both embedded (at separate positions) in contigs surrounded by known chromosomal genes. In eight, In*37* and *bla*_CTX-M-15_ were immediately adjacent on the same contig, sometimes surrounded by known chromosomal genes, and sometimes not, suggesting a variety of chromosomal and plasmid locations.

**Table 2.** χ^2^ analyses examining the association of *aac(6’)Ib-cr* carriage with other different genotypic factors

*aac(6’)Ib-cr* carriage versus:	χ^2^	Association	*P*
ST131	6.2	positive	0.013
ST1193	4.3	negative	0.038
ST38	1.9	positive	0.17
*bla*_CTX-M-15_ carriage	47.8	positive	<0.0001
*bla*_CTX-M-27_ carriage	5.0	negative	0.025
QRDR type A	8.7	positive	0.003
QRDR type B	3.5	negative	0.060
QRDR type C	3.0	negative	0.083

All comparisons had degrees of freedom = 1.

3GC-R ST131 urinary *E. coli* isolates commonly carry *bla*_CTX-M-15_ in this study region.^9^ Therefore, it was not surprising to find a positive association between *aac(6’)Ib-cr* carriage and ST131 and between *aac(6’)Ib-cr* carriage and QRDR type A (Table 2). However, of 32 FQ-R/3GC-S ST131 *E. coli*, only 4 carried *aac(6’)Ib-cr*, confirming *aac(6’)Ib-cr* was linked with *bla*_CTX-M-15_ and not with ST131.

In contrast, ST1193 isolates in this study were found mainly to be 3GC-S [*n *=* *28 (versus *n *=* *6 for 3GC-R); Figure 1] and there was a significant negative association between *aac(6’)Ib-cr* carriage and the isolate being ST1193 (Table 2). There was also a negative association between carriage of *bla*_CTX-M-27_ and carriage of *aac(6’)Ib-cr* (Table 2). These associations reinforce the conclusion that In*37* and *bla*_CTX-M-15_ are closely genetically linked in urinary *E. coli* in our study region. No other association tested was found to be statistically significant.

### Molecular epidemiology of FQ-R E. coli from dairy farms and analysis of possible farm–human sharing

We have recently reported a survey of resistant *E. coli* in faecal samples from 53 dairy farms in South-West England.^23^ Of 4145 faecal samples collected from vicinities near animals, 263 were positive for FQ-R *E. coli*, representing 49 of 53 farms surveyed.^23^ Of 42 farms located within a 50 × 50 km region, which included the homes of the people providing the urinary samples discussed above, 245 FQ-R *E. coli* isolates from this earlier study^23^ were taken forward for further analyses, representing 1 isolate from each positive sample. Multiplex PCRs showed that *aac(6’)Ib-cr* (found to be highly prevalent in the human urinary *E. coli* isolates) was entirely absent from the farm isolates and only nine isolates carried a PMQR gene (5 *qnrS*, 2 *qnrA* and 2 *qnrD)*.

**Figure 2.**
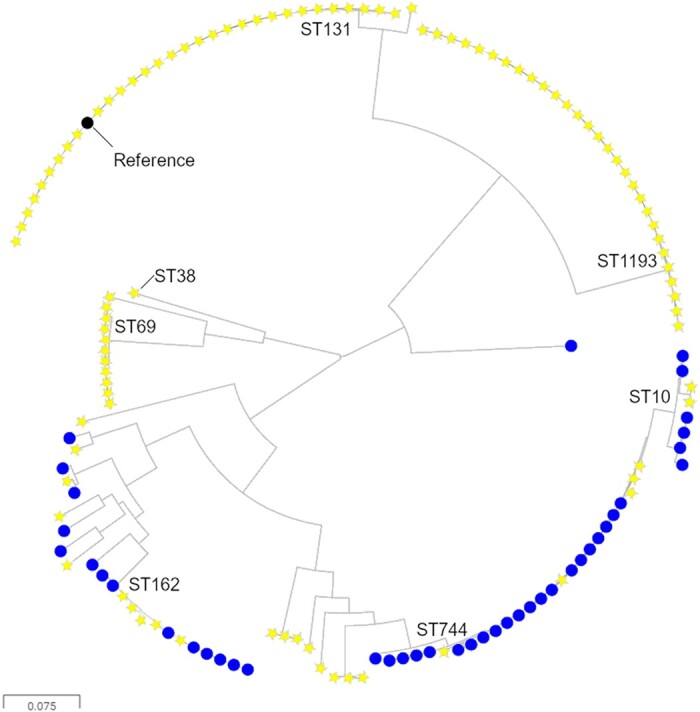
Maximum likelihood phylogenetic tree showing FQ-R, 3GC-S urinary isolates (yellow stars) and FQ-R cattle isolates (blue circles). Sequences were aligned against an ST131 reference (black node). Prevalent STs have been indicated. This figure appears in colour in the online version of *JAC* and in black and white in the print version of *JAC*.

**Figure 3.**
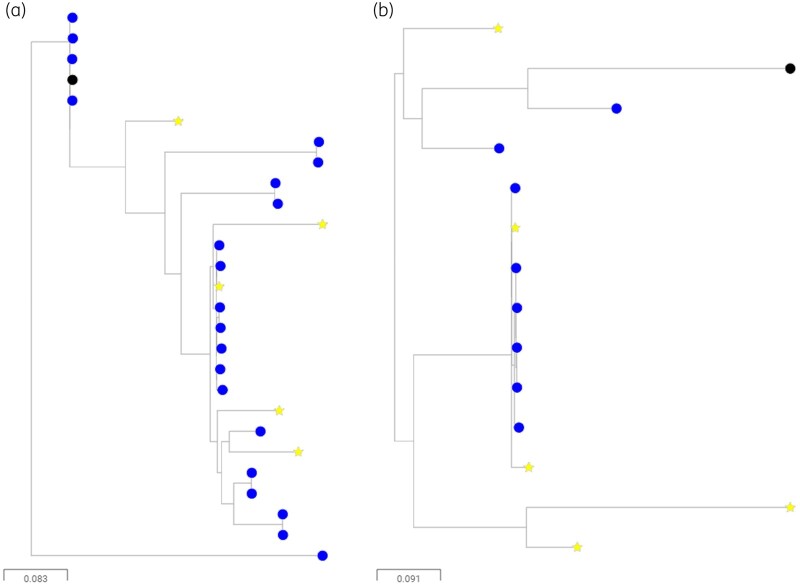
Maximum likelihood phylogenetic tree showing (a) ST744 isolates and (b) ST162 isolates also present in Figure 2. 3GC-S urinary isolates (yellow stars) and isolates collected from environments nearby to cattle housing (blue circles). Sequences were aligned against references of the same ST (black node). This figure appears in colour in the online version of *JAC* and in black and white in the print version of *JAC*.

WGS was performed for 42 FQ-R cattle-associated *E. coli*, which were selected so that at least 1 isolate represented each farm that was positive for FQ-R *E. coli* within the 50 × 50 km region. Nine different STs were identified, with dominance of ST744 (21/42 isolates), ST162 (8/42 isolates) and ST10 (5/42 isolates). ST162 and ST10 are QRDR type C , with three mutations as defined above (Table 1). ST744 also has these same three QRDR mutations, plus the addition of a distinctive *parC* mutation causing Ala56Thr. Because ST744 and ST162 were also common among FQ-R/3GC-S human isolates (Figure 1), we generated a phylogenetic tree to consider relationships between the cattle FQ-R and human FQ-R/3GC-S *E. coli* collected in parallel within our 50 × 50 km study area (Figure 2). This suggested close phylogenetic relationships among ST744 and ST162 isolates found on different farms, but, importantly, also among farm and human urinary isolates. Detailed trees generated using ST744 or ST162 reference genomes (Figure 3) confirmed this. The closest relationship between two human urinary isolates was 931 or 175 SNPs for ST744 and ST162, respectively, but the closest relationship between a human and a cattle isolate was 71 or 63 SNPs, respectively. The closest relationship between a pair of cattle isolates, each isolated on a different farm, was seven and three SNPs, respectively.

### Fluoroquinolone use positively associated with FQ-R E. coli on dairy farms and dry cow therapy negatively associated

We next considered factors that might influence the prevalence of FQ-R *E. coli* on farms. Our aim was to identify potential interventions that might reduce FQ-R *E. coli* on farms. In our recent paper, we showed that the odds of a sample being positive for FQ-R *E. coli* was significantly greater if the sample came from the environment of heifer calves.^23^ This is important because dairy heifer calves are normally reared on the farm as replacement milking cows, so factors associated with the increased carriage of FQ-R *E. coli* in calves could have a long-term effect on the whole farm. Accordingly, we performed a new risk factor analysis to identify management and ABU factors associated with the odds of finding FQ-R *E. coli*-positive samples in the environments of heifer calves.

Of 631 samples collected from the environments of heifer calves, 103 (16.3%) were positive for FQ-R *E. coli*. We identified two variables that were associated with sample-level positivity for FQ-R *E. coli*. One variable—the total usage of fluoroquinolones in the year the samples were collected—was positively associated with finding FQ-R *E. coli* in a sample (OR = 2.39, 95% credible interval = 1.01–6.02). Another variable—the percentage of cows within the herd dried off using any antibacterial dry cow therapy (a preparation inserted into the teats of pregnant cows thought to be at risk of developing mastitis between lactations or with existing subclinical infections) - was negatively associated with finding FQ-R *E. coli* in samples (OR = 0.24, 95% credible interval = 0.11–0.50). Full model outputs and model checking details are presented in Table S1, Table S2, Figure S1 and Figure S2 (available as Supplementary data at *JAC* Online).

## Discussion

We have shown in previous similarly powered work performed in parallel on the same study farms that there was no evidence of recent sharing of 3GC-R *E. coli* between farms and humans in the study region. It should be noted that we are only considering human isolates that have caused bacteriuria, not commensal carriage isolates.^21^^,^^22^ However, our studies agree with the findings of similarly motivated studies from other groups.^34–36^ We now report some evidence for sharing of FQ-R *E. coli* between dairy farms and humans, again when considering human isolates that have caused bacteriuria.

There is no definitive SNP distance cut-off to define ‘sharing’. A core genome SNP distance of <30 is commonly seen in phylogenetic analyses of Enterobacterales isolates that are confirmed to be part of an acute outbreak of foodborne illness^37^ and hospital studies frequently set a cut-off of <100 SNPs to define significant clonal relationships, which might be called ‘lineages’.^38^ Nonetheless, we show here that FQ-R *E. coli* farm–human isolate pairs differ by as little as 71 (ST744) or 63 (ST162) SNPs. This is at least suggestive of a situation where human and cattle isolates in this region intermingle. Whilst it is not possible to determine the direction, mechanisms or timing of transmission from this observation, important context also comes from our finding that the closest isolates from two different farms were only 3 and 7 SNPs apart for ST162 and ST744, respectively, and the closest pair of human isolates were 175 and 931 SNPs apart , respectively, for these two STs (Figure 3). It seems therefore that *E. coli* from these STs are readily moving between farms, less readily moving between farms and humans, and least readily moving between humans. This contrasts with work showing 3GC-R *E. coli* readily move between farms or between humans but very rarely move between farms and humans.^21^^,^^22^^,^^34–36^ More work is needed to establish the exact routes of transmission, but even the minor zoonotic potential of cattle-associated FQ-R *E. coli* hinted at here should act as a stimulus to reduce the prevalence of such bacteria on farms. This work also suggests potential ways to achieve that objective.

We have reported at a regional level that reducing fluoroquinolone use in primary care was associated with a reduction in the proportion of *E. coli*-positive community urine samples where the isolate was FQ-R.^15^ Accordingly, it was interesting to find that overall fluoroquinolone use at farm level was positively associated with the odds of finding FQ-R *E. coli*-positive faecal samples in the environments around dairy heifer calves. The implication is that reducing fluoroquinolone use on farms may well reduce the prevalence of FQ-R *E. coli*. Notably, in mid-2018, as our surveillance of resistant *E. coli* on study farms was ending, the use of fluoroquinolones was effectively stopped, except in the very rare instance where susceptibility testing confirmed that no other antibacterial treatment option was available. This was because of the introduction of a Red Tractor farm assurance scheme regulation, which is applicable to the vast majority of UK dairy farms.^19^

Our final key finding was that dry cow therapy use was associated with a reduction of FQ-R *E. coli* in heifer calves, which was unexpected. To explain this, we hypothesized that relatively few FQ-R *E. coli* from these farms were cross-resistant to the antibacterial ingredients of dry cow therapies. These antibacterials can be released in the colostrum and first milk from treated cows. Since this colostrum is usually fed to calves at birth,^39–41^ it is plausible that calves receiving colostrum from treated cows are protected from colonization by FQ-R *E. coli* due to the dose of antibacterial inadvertently received shortly after birth.

In support of this hypothesis, we calculated that 83% (in terms of weight of active ingredient) of the dry cow therapy anti-Gram-negative antibacterials used on study farms during the period of our project were cephalosporins or cloxacillin (3.44 kg). Framycetin (neomycin B) made up the remainder (0.69 kg). Notably, of the 42 FQ-R cattle *E. coli* isolates subjected to WGS, only 2 (4.8%) were resistant to both cephalosporins and cloxacillin, as inferred from WGS. Whilst genetically inferred framycetin resistance (presence of an *aph* gene) was more common among sequenced FQ-R cattle *E. coli* isolates (15/42, 36%), it was far less common among FQ-R isolates than among CTX-M β-lactamase-positive 3GC-R *E. coli* isolates from these same farms collected in parallel (109/135, 81% of isolates).^22^ Accordingly, there is evidence for suppression of FQ-R *E. coli* by dry cow therapy use, irrespective of active agent, because the FQ-R bacteria found on these farms are rarely resistant to the active agents used.

Much attention has been paid to reducing dry cow therapy use on dairy farms as a way of reducing total ABU.^39–41^ We would not suggest a shift away from this approach, because inappropriate use might increase the selection of cattle *E. coli* that are resistant to other antibacterials, as we have already reported for cefquinome dry cow therapy use and selection of CTX-M-producing *E. coli* in calves.^23^ Whilst cefquinome use also effectively stopped under the 2018 Red Tractor regulations,^19^ it is possible that switching to first-generation cephalosporin or cloxacillin dry cow therapy could maintain selection for CTX-M producers. Importantly, however, ∼50% of 3GC-R in *E. coli* on our study farms was caused by chromosomally encoded AmpC hyper-production,^21^^,^^22^ an enzyme inhibited by cloxacillin.^42^ We suggest, therefore, that a better alternative to cefquinome dry cow therapy might be cloxacillin, if all else is equal, which may help to reduce the prevalence of AmpC hyper-producer and FQ-R *E. coli*.

Overall, our One Health approach to investigating selection and transmission of critically important antibacterial-resistant *E. coli* within our study region identified rare but not insignificant farm–human sharing of FQ-R *E. coli*. Our results also highlight that reducing fluoroquinolone use on farms, whilst carefully selecting the most appropriate dry cow therapy active ingredient to match the ecology of resistance found on a particular farm, should most effectively reduce the prevalence of FQ-R *E. coli*. Our work certainly demonstrates the foresight of the recently introduced Red Tractor regulations designed to effectively eliminate use of highest-priority critically important antibacterials on dairy farms in the UK.^19^

## Supplementary Material

dkab310_Supplementary_Data
